# The Inhibition of the FGFR/PI3K/Akt Axis by AZD4547 Disrupts the Proangiogenic Microenvironment and Vasculogenic Mimicry Arising from the Interplay between Endothelial and Triple-Negative Breast Cancer Cells

**DOI:** 10.3390/ijms241813770

**Published:** 2023-09-06

**Authors:** Gabriela Morales-Guadarrama, Edgar A. Méndez-Pérez, Janice García-Quiroz, Euclides Avila, María J. Ibarra-Sánchez, José Esparza-López, Rocío García-Becerra, Fernando Larrea, Lorenza Díaz

**Affiliations:** 1 Departamento de Biología de la Reproducción Dr. Carlos Gual Castro, Instituto Nacional de Ciencias Médicas y Nutrición Salvador Zubirán (INCMNSZ), Vasco de Quiroga No. 15, Belisario Domínguez Sección XVI, Tlalpan, Ciudad de México 14080, Mexico; gabriela.mguadarrama@gmail.com (G.M.-G.);; 2 Unidad de Bioquímica Dr. Guillermo Soberón Acevedo, Instituto Nacional de Ciencias Médicas y Nutrición Salvador Zubirán (INCMNSZ), Vasco de Quiroga No. 15, Belisario Domínguez Sección XVI, Tlalpan, Ciudad de México 14080, Mexico; 3 Departamento de Biología Molecular y Biotecnología, Instituto de Investigaciones Biomédicas, Universidad Nacional Autónoma de México, Av. Universidad 3000, Coyoacán, Ciudad de México 04510, Mexico; 4 Programa de Investigación de Cáncer de Mama, Instituto de Investigaciones Biomédicas, Universidad Nacional Autónoma de México, Av. Universidad 3000, Coyoacán, Ciudad de México 04510, Mexico

**Keywords:** co-culture, tubulogenesis, vascular mimicry, breast cancer, angiogenesis array, AZD4547, LY294002

## Abstract

Vasculogenic mimicry (VM), a process in which aggressive cancer cells form tube-like structures, plays a crucial role in providing nutrients and escape routes. Highly plastic tumor cells, such as those with the triple-negative breast cancer (TNBC) phenotype, can develop VM. However, little is known about the interplay between the cellular components of the tumor microenvironment and TNBC cells’ VM capacity. In this study, we analyzed the ability of endothelial and stromal cells to induce VM when interacting with TNBC cells and analyzed the involvement of the FGFR/PI3K/Akt pathway in this process. VM was corroborated using fluorescently labeled TNBC cells. Only endothelial cells triggered VM formation, suggesting a predominant role of paracrine/juxtacrine factors from an endothelial origin in VM development. Via immunocytochemistry, qPCR, and secretome analyses, we determined an increased expression of proangiogenic factors as well as stemness markers in VM-forming cancer cells. Similarly, endothelial cells primed by TNBC cells showed an upregulation of proangiogenic molecules, including FGF, VEGFA, and several inflammatory cytokines. Endothelium-dependent TNBC-VM formation was prevented by AZD4547 or LY294002, strongly suggesting the involvement of the FGFR/PI3K/Akt axis in this process. Given that VM is associated with poor clinical prognosis, targeting FGFR/PI3K/Akt pharmacologically may hold promise for treating and preventing VM in TNBC tumors.

## 1. Introduction

In cancer, tumor growth and dissemination depend on adequate access to the host blood supply through neo-vascularization, a process comprising the formation of microvascular networks capable of perfusion that develop in response to local poor blood flow or ischemia [1]. Tumor neo-vascularization can be achieved through different mechanisms, including (a) angiogenesis, an endothelial-dependent event where new vessels arise from preexistent ones; (b) mosaic vessel formation, in which endothelial cells (ECs) and cancer cells intermingle to form vessels; or (c) vasculogenic mimicry (VM), where cancer cells undergo phenotypical changes allowing them to secrete extracellular matrix (ECM) components to form tubular structures as blood channels [1,2,3].

Notably, cancer cells capable of forming VM exhibit high plasticity, which enables them to transdifferentiate into endothelial-like cells co-expressing endothelial, embryonic/stem, and tumor markers. These cells also show aberrant expression of specific glycoproteins such as vascular endothelial cadherin (VE-cadherin) and CD133 [4,5,6].

Since a lack of oxygen acts as a VM driver, VM can develop after an antiangiogenic treatment or in hypoxic regions of highly aggressive tumors, including breast cancer, with increased frequency in the triple-negative breast cancer (TNBC) phenotype [7,8]. TNBC tumor cells are known to secrete factors and exosomes that induce tumorigenic features in neighboring normal cells by changing their metabolic program to become tumor-supportive [9]. However, little is known about the participation of ECs and stromal cells (SCs) in the microenvironmental changes and reprogramming of cancer cells that enable them for VM formation. Moreover, even if previous studies have shown the ability of ECs to induce VM in breast cancer [10,11,12,13], there is limited information so far on the factors provided by these cells that are capable of inducing VM and the mechanisms involved. Therefore, in this study, we aimed to explore the capacity of ECs and SCs to induce VM in two different TNBC cell lines using an in vitro co-culture model that allows us to study their close interaction. In addition, knowing that the loss of equilibrium between the activators and repressors of vasculogenesis in the tumor microenvironment involves modifications of the secretome and the ECM, which can lead to the activation of both VM and angiogenesis [8], herein, we also characterized the endothelial-dependent pro-vasculogenic signature occurring in co-cultures (CCs) of ECs and cancer cells. In an oncogenic setting, both ECs and cancer cells can undergo activation not only of the angiogenic switch but also of the metabolic switch, increasing glycolysis to satisfy the high energy demand, resulting in a complete metabolic signature transformation [13,14]. In this regard, hexokinase 2 (HK2), a rate-limiting glycolytic enzyme that turns on the metabolic switch for cancer progression and vascularization, can be activated by a variety of proangiogenic molecules, including hypoxia-inducible factor-1α (HIF1A), vascular endothelial growth factor (VEGFA) and some members of the fibroblast growth factor (FGF) family [15,16]. Among the 22 members of the FGF family, 18 interact with the tyrosine kinase receptors FGFR1, FGFR2, FGFR3 and FGFR4, regulating diverse cellular functions such as the enhancement of cellular proliferation, motility, invasiveness, metastasis and angiogenesis [17]. FGFs are released into the tumor microenvironment when liberated from the ECM or may be secreted by cancer cells and/or other cell types, thereby enriching the tumor microenvironment secretome [18]. The FGF–FGFR axis is one of the most relevant proangiogenic signaling mediators, suggesting that it can play a key inductive role in VM formation by activating both ECs and cancer cells [19,20]. Indeed, the angiogenic pathway in TNBC cells is known to be closely related to VM [21]. Notably, the binding of FGFs to FGFRs activates major oncogenic intracellular signaling pathways, including the phosphatidylinositol 3-kinase/Akt (PI3K-AKT) pathway [22,23]. Therefore, in this study, we also aimed to explore the possibility of blocking VM in TNBC cells through inhibiting FGF/FGFR signaling using AZD4547, a potent and selective inhibitor of FGFRs [24], and LY294002, a PI3K inhibitor.

## 2. Results

### 2.1. Endothelial Cells, but Not Stromal Cells, Enable VM Formation by TNBC Cells, Which Undergo Phenotypic and Morphological Changes during the Formation of Tubular-like Structures

**Figure 1:**
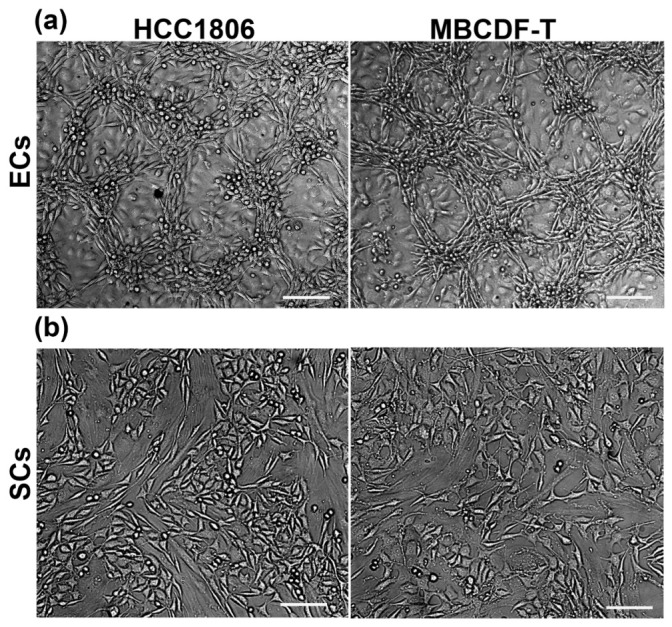
Endothelial cells, but not stromal cells, enabled VM formation by TNBC cells. EA.hy926 endothelial cells (ECs), but not N30 stromal cells (SCs), promoted the formation of vasculogenic mimicry by two different TNBC cell lines (HCC1806 or MBCDF-T). Two tumor microenvironment cell lineages, ECs (**a**) or SCs (**b**), were co-seeded with TNBC cells for 48 h, and the formation of tubular-like three-dimensional structures was observed only with ECs and TNBC co-cultures (**a**). Live cells were photographed, and representative bright field images are shown. Pictures are magnifications from 10× images, scale bar = 100 μm.

Previously, we and others have shown the paracrine contribution of ECs in tumor cell behavior, including TNBC VM formation [10,12,25,26]. In this study, our aim was to gain further insight into the contribution of cellular components of the tumor microenvironment in TNBC VM induction, focusing on identifying the participation of ECs and SCs in this process. To investigate this, two TNBC cell lines, MBCDF-T and HCC1806, were independently co-incubated with SCs (N30 cells) or ECs (EA.hy926) for 48 h (Figure 1). As depicted, only the co-culture of cancer cells with ECs showed VM formation. The co-culture of HCC1806 or MBCDF-T cells with ECs resulted in a complete cytoarchitectural change, displaying two noticeable focal planes in the dish: one below forming cellular packages in a monolayer, and the second one above the first one, arranged in tridimensional tubular-like structures engaged in networks (Figure 1, upper panels). In contrast, in the case of SCs-TNBC CCs, the cells were distributed in a monolayer within a single focal plane and showed mesenchymal-like features (Figure 1, lower panels). Therefore, ECs, but not SCs, supported cancer cells’ transdifferentiation, enabling them to form tubular networks. The morphological modifications that each cell lineage showed when co-cultured included a reduced nuclear/cytoplasmic proportion and a needle-like shape in TNBC cells, while EA.hy926 cells seemed enlarged in their cytoplasmic proportion and displayed a bigger nuclear size than TNBC cells. Some cancer cells showed a round shape, probably related to recent mitosis.

**Figure 2:**
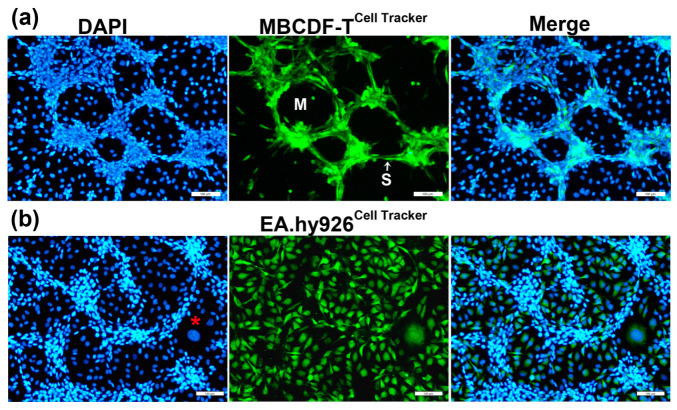
TNBC cells form the tubular structures in endothelial–breast cancer co-cultures. ECs and TNBC cells co-cultures resulted in VM formation rather than endothelial angiogenesis. (**a**) MBCDF-T cells were labeled with a green cell tracker and then co-cultured with unlabeled ECs (EA.hy926) for 48 h. As depicted, exclusively TNBC cells were involved in the tubular-like network, displaying a spindle-like shape and conforming segments (S) and meshes (M). (**b**) EA.hy926 cells were labeled with the green cell tracker before co-culturing them with unlabeled MBCDF-T cells. The nuclei were counterstained with DAPI (blue channel). Giant, flattened ECs characterized by a large nucleus may be found in the co-cultures (red asterisk). Representative images were obtained via epifluorescence microscopy. Magnifications from 10× pictures are shown. Scale bar = 100 μm.

To confirm that the tubular networks in TNBC-ECs CCs were formed by cancer cells and not by the ECs themselves, we labeled TNBC cells (MBCDF-T) with a fluorescent green cell tracker and then co-cultivated them with unlabeled EA.hy926 cells (Figure 2a). As depicted in Figure 2a, only the green-labeled cancer cells were involved in VM formation in the co-culture, ruling out endothelial angiogenesis. Moreover, when we conducted the opposite experiment, labeling ECs with the cell tracker instead of TNBC cells and co-cultured them, we found that ECs were clearly arranged below the tubular networks in the lower focal plane (Figure 2b). In some cases, ECs seemed to rearrange themselves into patches, leaving spaces for the tubular cancer structures. However, in other instances, their configuration suggested that they acted like scaffolds for the tridimensional TNBC assemblies, probably providing ECM and mechanical support for TNBC-VM. In the TNBC-3D networks, two different VM structures were identified, as previously reported [10], and as shown in Figure 2a: segments, defined as cords or branches formed by spindle-shaped TNBC cells, and meshes, represented as the enclosed areas surrounded by the segments, showing underlying ECs visible via DAPI-staining of their nuclei (Figure 2a).

**Figure 3:**
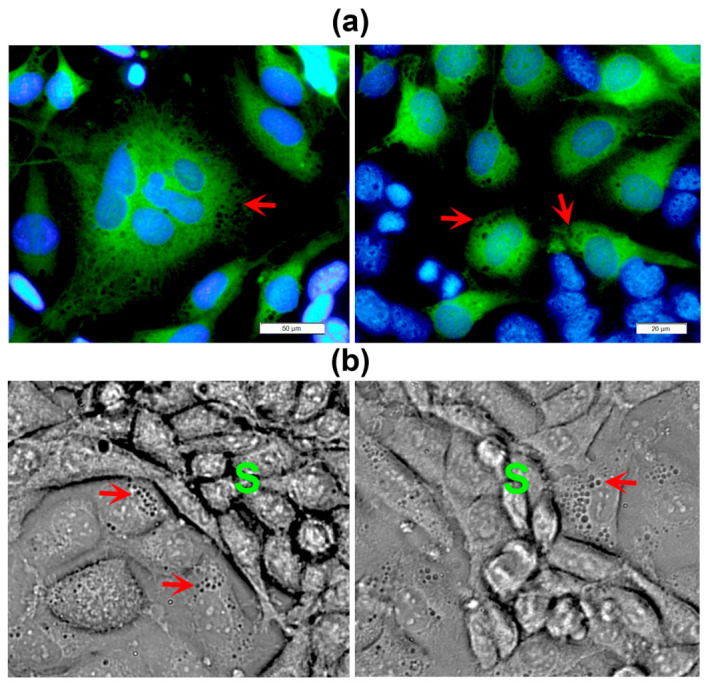
In close areas neighboring the VM segments endothelial cells show a highly vacuolated cytoplasm. (**a**) EA.hy926 cells were labeled with a green cell tracker before co-culturing them with unlabeled MBCDF-T cells for 48 h. Nuclei were counterstained with DAPI (blue channel). Non-green cells represent cancer cells. Magnifications from 20× and 40× images; scale bar = 50 μm and 20 μm in left and right panel, respectively. (**b**) Live HCC1806 cells co-cultured with EA.hy926 cells during 48 h were photographed. Red arrows show vacuoles in the endothelial cells neighboring the VM segments (S). Cancer cells appear smaller and elongated, with a low cytoplasm/nucleus ratio, while endothelial cells look bigger and flattened, with large nuclei and greater cytoplasm/nucleus ratio. Magnifications from 40× bright field images are shown.

Interestingly, the ECs located in close contact with the VM segments exhibited a highly vacuolated cytoplasm (Figure 3), suggesting their active participation in the conformation of the VM meshes, probably through providing necessary ECM components and/or pro-VM factors from endothelial origin. Occasionally, we could also observe giant, flattened ECs characterized by a large nucleus (Figure 2, red asterisk) or containing multiple nuclei with an atypical shape (Figure 3a). These cells could present a disrupted nuclear membrane, showing features of cell death. We named these cells modified multinucleated/multivacuolated endothelial cells (MMECs). We believe that MMECs may participate in VM structure formation. As judged by the green-tracker studies, MMECs appear to be modified ECs (Figure 3a). Nonetheless, cancer multinucleated giant cells could also be identified, starting to form as early as 2–4 h after co-seeding both cell types, and were also found in TNBC monocultures.

Taken together, these results indicate that ECs, but not SCs, are able to induce TNBC-VM formation when co-cultured, without engaging themselves in the tridimensional tubular structures but providing VM drivers and ECM components to support them.

### 2.2. Co-Cultured Cells Differentially Express Markers of VM and Endothelium

**Figure 4:**
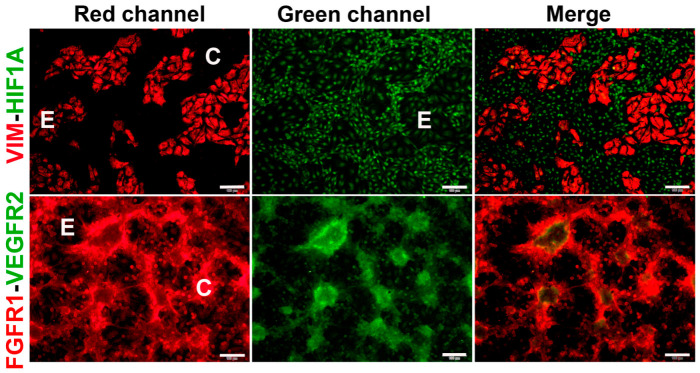
Co-cultured TNBC cells and endothelial cells differentially express markers of VM and endothelium. HCC1806 cells and EA.hy926 cells were co-cultured for 48 h. Then, co-cultures were fixed and further processed for immunocytochemistry. Each slide was incubated with two primary antibodies, one made in mice and one in rabbits. Incubations were undertaken overnight at 4 °C. Mice-made primary antibodies were detected using a secondary goat anti-mouse-Cy3 antibody (red channel, left panels). Rabbit antibodies were detected with a goat anti-rabbit-FITC (green channel, middle panels). The right panels show merged pictures. As a guide, the letter “C” depicts the location of cancer cells in the segments of VM structures, while the letter “E” depicts the endothelial cells position. Cell images were captured using a conventional fluorescence microscope. Representative 10× images are shown. Scale bar = 100 μm. Vimentin—VIM; hypoxia-inducible factor-1α—HIF1; vascular endothelial growth factor receptor 2—VEGFR2; fibroblast growth factor receptor 1—FGFR1.

**Figure 5:**
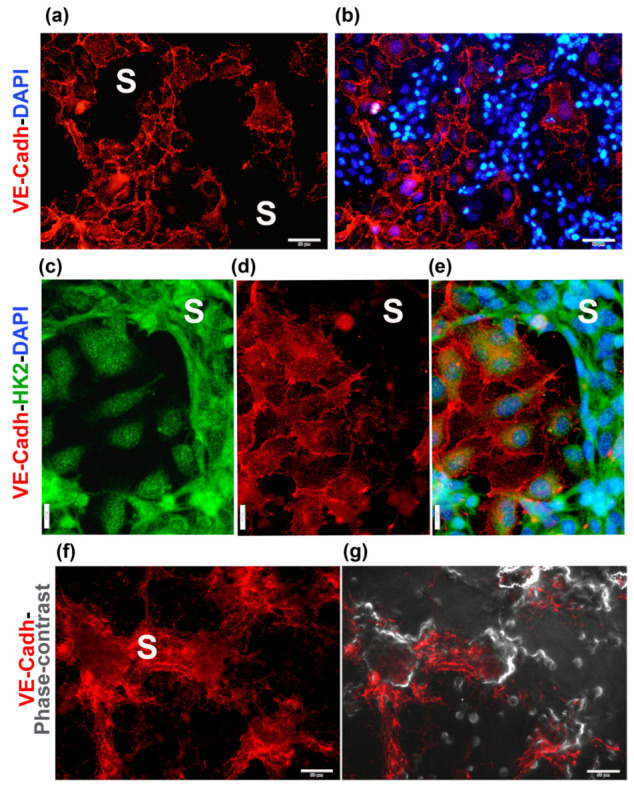
VE-cadherin and HK2 are differentially localized in the endothelial and cancer components of the co-cultures. HCC1806 cells and EA.hy926 cells were co-cultured for 48 h. Then, co-cultures were fixed and further processed for immunocytochemistry. Mouse anti-VE-cadherin (VE-Cadh) and rabbit anti-hexokinase 2 (HK2) were used as primary antibodies. Secondary antibodies were goat anti-mouse-Cy3 (red channel) and goat anti-rabbit-FITC (green channel). Junctional VE-Cadh is seen in the endothelial component of the co-cultures, while it is scarcely expressed in TNBCs (**a**,**b**,**d**,**e**). In certain portions of the preparations, nuclear and membrane localization of VE-Cadh in TNBC cells forming the vasculogenic mimicry segments (S) and branch intersections can also be clearly seen (**f**,**g**). DAPI blue staining is seen in (**b**,**e**). Cell images were captured using a conventional fluorescence microscope (**a**–**f**), while (**g**) is the merged image of (**f**) and the corresponding phase contrast photograph. Magnification is as follows: (**a**,**b**,**f**,**g**) use 20× magnification (scale bar = 50 μm); (**c**–**e**) are 40× images (scale bar = 20 μm).

It is well established that tumor cells undergoing VM develop several metabolic changes in response to environmental stressors such as hypoxia and other VM drivers. These changes include metabolic reprogramming involving the upregulation of HK2 and the expression of endothelial markers such as VE-cadherin [4,27,28,29]. Other markers associated with vasculogenesis/VM include HIF1A, vimentin and the receptors FGFR1 and VEGFR2 [15,30]. Therefore, we performed immunocytochemistry studies to investigate the status of these proteins in each cell lineage conforming the CCs. As depicted in Figure 4, each cell lineage in co-culture displays a specific expression and localization pattern for the protein markers. In particular, vimentin was highly expressed in the cytoskeleton of ECs (Figure 4, upper panel, red channel), a feature associated with cell–ECM focal adhesions and endothelial sprouting [31,32]. Similarly, VE-cadherin, the major endothelial adhesion molecule, was highly expressed in ECs, localized in the membranes at the junctions between the cells, with low diffuse expression in the cytosol (Figure 5, upper and middle panels, red channel). This localization of vimentin and VE-cadherin was observed in ECs both in monoculture (Supplementary Figure S1) and co-culture (Figure 4 and Figure 5). In comparison, TNBC cells showed weak vimentin cytoplasmic/nuclear expression in monoculture (Supplementary Figure S1), while it was virtually undetected in TNBC cells arranged in the VM segments of the CCs (Figure 4 upper panel). As for VE-cadherin, low cytosolic and nuclear expression was identified in monocultures of TNBC cells (Supplementary Figure S1). However, when co-culturing TNBCs with ECs, nuclear and membrane localization of VE-cadherin could be observed at higher magnification in the segments and branch intersections of some TNBC VM segments (Figure 5f,g, red channel). Nuclear HIF1A was found in both ECs and TNBC cells monocultures (Supplementary Figure S1, green channel, first two rows) and CCs, with a more robust expression pattern in VM forming cells (Figure 4 upper panel, green channel). On the other hand, while in EC monocultures the HK2 signal was low and found mainly in the nuclear compartment (Supplementary Figure S1), in TNBC monocultures HK2 signal was high and localized with a diffuse pattern in cell nuclei, and enriched at the cytoplasmic compartment (Supplementary Figure S1). Notably, when both cell lines were co-incubated, the expression of HK2 increased mainly in the cells engaged in VM (Figure 5, middle panel, green channel).

Regarding the receptors mediating angiogenic signals, we investigated the expression and localization profiles of FGFR1 and VEGFR2. As depicted in Supplementary Figure S1, while monocultures showed practically undetectable expression of both receptors in ECs, a relatively high nuclear and cytoplasmic abundance of VEGFR2 and FGFR1, respectively, was observed in TNBC cells (Supplementary Figure S1). However, when co-cultured, both receptors were more strongly expressed in the cells engaged in the tubular-like structures, showing a lower signal in the underlying ECs (Figure 4, lower panel).

**Figure 6:**
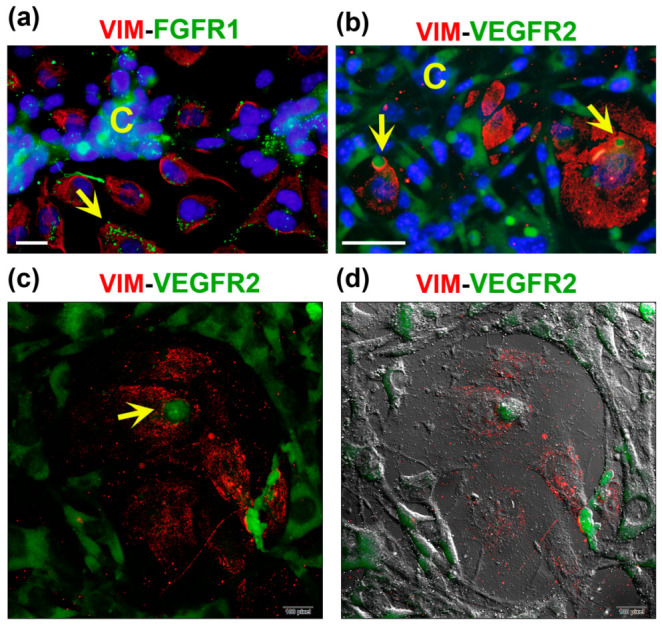
FGFR1 and VEGFR2 are localized in vesicles and vacuoles of endothelial cells in CCs. MBCDF-T and EA.hy926 cells were co-cultured for 48 h, followed by fixation and immunocytochemistry processing. (**a**) The primary antibodies mouse-anti-vimentin (VIM) and rabbit-anti-FGFR1 were co-incubated overnight at 4 °C. (**b**) Mouse-anti-VIM along with rabbit-anti-VEGFR2 were co-incubated overnight. The secondary antibodies used were goat anti-mouse-Cy3 (red) and goat anti-rabbit-FITC (green). In (**a**,**b**), DAPI-containing mounting media was used for nuclei staining (blue). In (**a**) the yellow arrow indicates the presence of small but plenty of FGFR1-containing vacuoles (green) in an endothelial cell, identified via VIM expression (red). The letter C shows cancer cells in the nearest VM segment. The photo is a magnification from a 40× image acquired with a conventional fluorescence microscope, scale bar = 20 μm. In (**b**), C depicts VEGFR2-positive cancer cells (green) in VM segments that surround endothelial VIM-expressing cells (red), which contain vesicles with VEGFR2 in green color (yellow arrows). Magnification from a 20× photograph acquired with a conventional fluorescence microscope (scale bar = 50 μm). (**c**,**d**) Confocal microscopy images of the same immunocytochemistry preparation as in (**b**), showing VIM-positive endothelial cells and VEGFR2-positive MBCDF-T cells. The arrow indicates a structure suggesting a VEGFR2-positive vacuole (green) at 60× magnification. (**d**) Confocal phase contrast photography merged with the image in (**c**).

Notably, at a higher magnification, we observed the expression of FGFR1 and VEGFR2 in vesicles/vacuoles within the vimentin-positive MMECs, with increased number of these subcellular structures in the cells located near the branches of the VM networks (Figure 6a,b). In these images, vimentin was used to identify ECs (Figure 6, red channel), while both receptors can be visualized in the green channel. The increased expression of FGFR1 and VEGFR2 in cancer VM-forming cells, along with their specific localization pattern within the MMECs of the CCs, suggest an important role for these receptors in intercellular communication, possibly contributing to the formation of VM structures.

### 2.3. Co-Cultured Endothelial and TNBC Cells Modify the Equilibrium of Proangiogenic and Anti-Angiogenic Molecules in the Secretome, Favoring a Pro-VM Microenvironment

**Figure 7:**
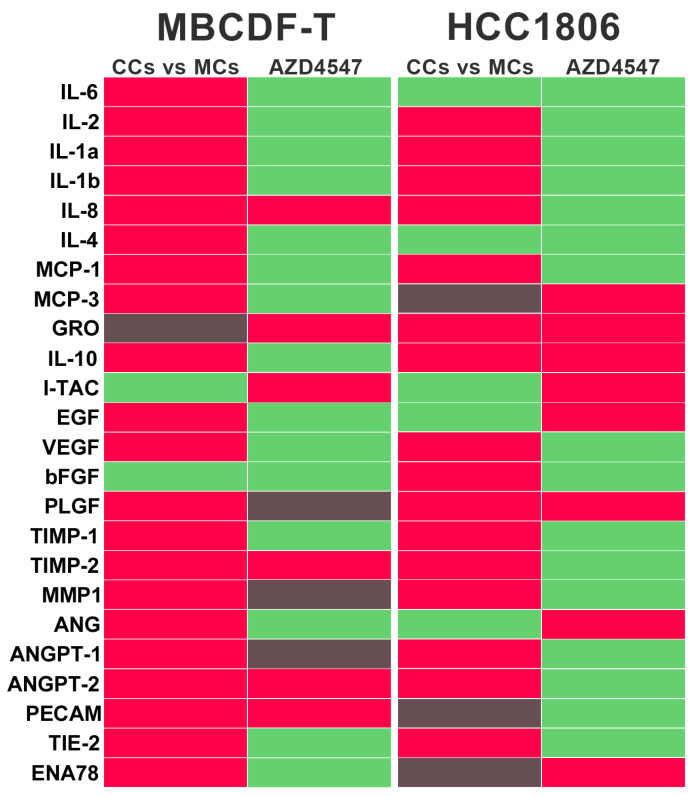
The interaction between endothelial and TNBC cells induces a proangiogenic secretome, which is suppressed by AZD4547. Results from the angiogenesis antibody array analysis showing the graphical color representation of the shift in the abundance of each factor in the secretome from co-cultures (CCs) after comparing against the secretome from endothelial monocultures (MCs). This effect is shown in the left column of the two TNBC cell lines, MBCDF-T and HCC1806, respectively (CCs vs. MCs). The change in the abundance of the angioregulatory factors in the secretome from CCs treated with the FGFR inhibitor AZD4547, compared against the CM of untreated CCs, is shown in the right column of each cell line (AZD4547). The red color indicates a rise in the abundance of each factor, whereas the green color indicates downregulation. The gray color depicts no change. As depicted, co-culturing endothelial cells with either TNBC cell line resulted in a general upregulation of proangiogenic factors as compared with the secretome from monocultured endothelial cells, while AZD4547 suppressed this effect.

By modifying the microenvironment components of the tumor niche, cancer cells can recruit several types of cells from the host, including those with an endothelial, stromal or immune lineage [33,34,35,36]. However, there is limited knowledge about the reciprocal communication between cellular components promoting VM. Therefore, we conducted an analysis of vasculogenesis-related factors in the secretome (conditioned media, CM) of EA.hy926 and TNBC cells mono and CCs using a commercial angiogenesis antibody array. Surprisingly, in the CM from TNBC cells, many targets in the array were either undetected or present at very low levels, particularly in MBCDF-T cells. In contrast, the secretome from ECs was relatively more enriched in the analyzed components (Supplementary Table S1). Among the proangiogenic factors more highly detected in the CM from ECs (considering a normalized densitometric value > 0.01) were interleukin (IL)-8, IL-6, IL-4, monocyte chemoattractant protein-1 (MCP1), FGF, angiogenin (ANG), epithelial-neutrophil activating peptide (ENA78) and placental growth factor (PLGF). In comparison, among the anti-angiogenic factors, a high abundance of the tissue inhibitors of metalloproteinases (TIMP)- 1 and TIMP-2 and C-X-C motif chemokine ligand 11 (CXCL11, I-TAC) and angiopoietin-2 (ANGPT2) was found (Supplementary Table S1). The combination of these factors suggested an equilibrium between pro- and anti-angiogenic molecules, possibly favoring a deactivated angiogenic switch, explaining the lack of angiogenesis in monocultured ECs. However, when both cell lineages ECs and cancer cells were co-cultured, the abundance of several proangiogenic factors increased compared to the CM of monocultured ECs, including several cytokines, chemokines, and growth factors (Figure 7, red color and Supplementary Table S1). Notably, matrix metalloproteinase (MMP)-1 and VEGF were highly induced, together with IL-8 and MCP-1. FGF was also induced, but only in the EC-HCC1806 CCs. On the other hand, whereas the abundance of TIMPs was increased in the CM from CCs (Figure 7 red color, and Supplementary Table S1), the anti-angiogenic molecule I-TAC was considerably reduced, way below the threshold of 0.01 in both TNBC cells CCs (Figure 7, green color, and Supplementary Table S1). In a similar way as observed with comparisons against CM from ECs, when compared with the CM from monocultured TNBCs, many pro-VM factors were increased when both cell lineages were co-incubated, especially MMP1, ANGPT-1 and inflammatory cytokines, while I-TAC was downregulated in HCC1806 (Supplementary Table S1). Considering this, the results suggest a shift in the balance of proangiogenic factors and endogenous angiogenesis inhibitors, promoting activation of the VM switch when both ECs and TNBC cells were co-incubated.

**Figure 8:**
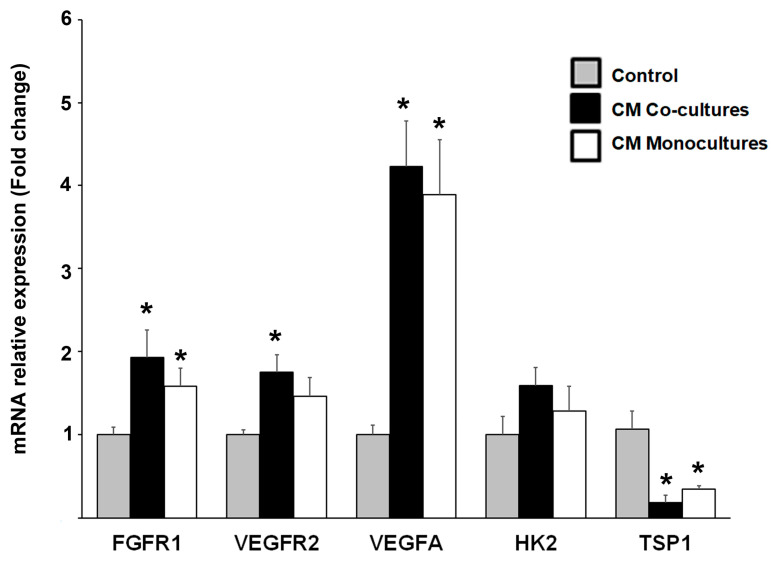
Relative mRNA expression levels of proangiogenic molecules in endothelial cells exposed to conditioned media from co-cultures and monocultures. The relative gene expression of *FGFR1*, *VEGFR2*, *VEGFA*, *HK2* and *THBS1* was analyzed via RT-qPCR in EA.hy926 endothelial cells (ECs) incubated for 24 h with the conditioned media (CM) from monocultured ECs (control), TNBC-ECs co-cultures or CM from TNBC monocultures. Bars in gray represent the mean normalized values ± SEM of the relative expression of targets assessed in ECs monocultures, which was set to one. Black and white bars represent mean values ± SEM of normalized expression of targets assessed in EC exposed to CM from co-cultures or CM from TNBC monocultures, respectively. GAPDH was used as a housekeeping gene for relative expression normalization. Data were obtained from at least three independent experiments. * *p* < 0.05 vs. control.

At this point, the evidence suggests that the endothelial lineage plays a crucial role in engaging TNBC cells into VM formation; however, given the pattern of VEGFR2 and FGFR protein expression in MMECs located in close contact with the VM segments, we wondered if TNBC cells induced ECs reprogramming through reciprocally interacting with them. So, to investigate this, we studied the regulation of mRNA expression of selected markers in ECs exposed to the secretome from TNBC monocultures and CCs. For this, we incubated EA.hy926 cells in the presence of either CM and determined the relative mRNA expression of *FGFR1*, *HK2*, *VEGFR2*, *VEGFA* and the negative regulator of angiogenesis thrombospondin-1 (*THBS1*). As depicted in Figure 8, and as compared with ECs incubated with control media, CM from CCs and/or from TNBC monocultures upregulated the gene expression of these angioregulatory factors, except for *THBS1*, which was significantly downregulated, corroborating the promotion of a proangiogenic profile in ECs exposed to CM from cancer cells (Figure 8).

On the other hand, incubation of MBCDF-T cells with the CM from CCs resulted in the induction of the stemness markers *OCT4*, *SOX2*, *KLF4* and *MYC* (Table 1). Of note, the gene expression of *SOX2* and *KLF4* was only detected after exposure of cells to CM. It is noteworthy mentioning that the stemness phenotype has been shown to significantly contribute to the malignant biological behavior of dormant polyploid giant cancer cells, prompting them to form VM [37]. It is worth mentioning that the exposure of monocultures (ECs or TNBC cells) to the CM of CCs did not result in the formation of tridimensional tubular structures, probably due to the lack of ECM factors produced by the physical interaction between the two cell lineages. However, changes in morphology could be noticed, particularly in the polarity of the cells.

All considered, the results suggest that through interacting with TNBC cells, ECs act as the main contributors of soluble factors into the CC secretome to establish a vasculogenic microenvironment suitable for VM development through inducing in TNBC cells stem-cell-like characteristics. However, ECM components produced by the interplay of ECs and TNBC cells also play an important role in this process.

### 2.4. VM Capacity of TNBC in Co-Cultures Is Regulated by FGFR1/PI3K/Akt Pathway

The FGF–FGFR axis is one of the most relevant proangiogenic signaling mediators activating both ECs and cancer cells and is closely involved in VM formation [19,20,38]. Considering the latter, together with the high FGFR1 expression observed in VM-engaged TNBC cells, and given that FGFR activation triggers the PI3K-Akt pathway [22,23], we explored the effect of blocking this pathway upon VM formation.

**Figure 9:**
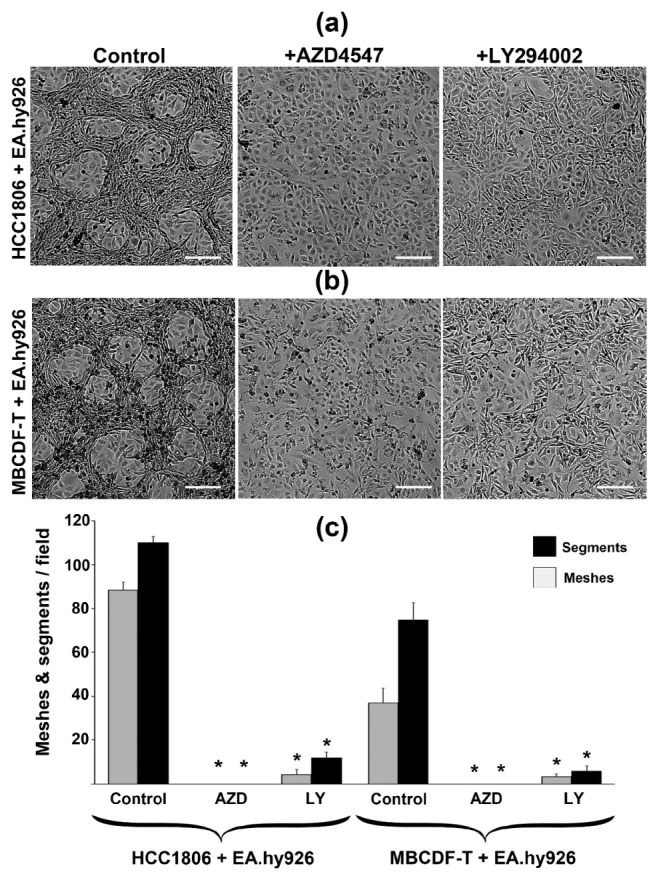
The FGFR1/PI3K/Akt pathway is involved in TNBC cells VM capacity. The interaction of EA.hy926 cells with two different TNBC cell lines (**a**) HCC1806 or (**b**) MBCDF-T resulted in VM formation (Control). Incubation of these co-cultures in the presence of 5 µM AZD4547 or 6 µM LY294002 for 2 days prevented the formation of tubular-like structures. (**c**) Evaluation of the effect of each compound on VM morphometric parameters. The mean number of segments ± SEM is shown in black bars, while the mean number of meshes ± SEM is in gray bars. In (**a**,**b**), representative amplifications from 4× bright field images are shown. Scale bar is 100 μm. Two observers counted the segments and meshes per visual field in hot spots of at least three 4× magnification images. * *p* < 0.05 vs. control.

First, to confirm the involvement of FGFR signaling in the VM capacity of TNBC cells, we used AZD4547, a selective FGFR inhibitor. EC–TNBC CCs were exposed to 5 µM AZD4547 for 2 days. As shown in Figure 9, the inhibition of FGFRs with this compound disrupted the formation of tubular-like structures, while untreated control CCs readily formed VM. To identify downstream VM-associated signaling pathways, we blocked the PI3K–Akt axis by using the PI3K-inhibitor LY294002. As depicted in Figure 9, the presence of 6 µM LY294002 almost completely abolished the formation of tube-like structures. Morphological changes were observed in the AZD4547-treated TNBC cells, specifically, shrinking and a more spherical form, contrasting with the spindle-shaped form observed when forming tubular like-structures. In contrast, there were no apparent cellular shape changes in the co-cultured ECs (Figure 9).

Thus, both FGFR and PI3K inhibitors displayed similar VM-disrupting effects, suggesting that the activation of the FGFR/PI3K–Akt axis is responsible for triggering TNBC cells VM capacity and the ability of ECs to promote an angiogenic environment.

Further, to explore which secretome components were being affected by AZD4547 in the CCs, we compared the angiogenesis array results obtained with CCs in the presence or absence of this FGFR-inhibitor. As depicted in Figure 7 and Supplementary Table S1, several pro-VM/angiogenic factors that had been upregulated through co-culturing ECs and TNBC cells were readily downregulated by AZD4547, considering at least one TNBC cell line (Figure 7, green color, and Supplementary Table S1). Notably, this effect was observed for various cytokines, chemokines and growth factors, including the most abundant ones: MMP-1, VEGF, IL-8 and MCP-1. Interestingly, the anti-angiogenic I-TAC, which had been downregulated in the CCs, was upregulated by AZD4547 (Figure 7, green color, and Supplementary Table S1).

## 3. Discussion

Vasculogenic mimicry is an alternative vascularization mechanism that aggressive tumors develop to obtain oxygen and nutrients either in response to specific tumor microenvironment-derived factors or after exposure to therapeutic treatments [1,2,3,7,8]. Although VM is clinically relevant due to its association with poor prognosis and metastasis, the available in vitro models to study this process are scarce and limited. These models are generally restricted to the interaction between cancer cells and commercially available ECMs, which contain a standardized set of extracellular matrix proteins and growth factors. Herein, we characterized a simple CC model to study VM in vitro that considers the interplay between cancer and endothelial cellular components, mimicking the in vivo tumor microenvironment more accurately. The CCs allow exploring the physical interaction between TNBC cells and ECs, which are known to stimulate VM in breast cancer through paracrine signaling [10,25], while at the same time consider the mixture of VM-driving factors produced by their mutual communication. Numerous studies have previously reported models of tumor cells-ECs interaction; however, they were used to address angiogenesis, ECs proliferation, or cross-talk between cancer cells and ECs, but not VM formation [13,39,40,41,42]. Nonetheless, the paracrine effects of ECs on tumor cell behavior have been described [12]. While in our study the ECs in monoculture underwent polarity changes after exposure to CM from TNBC cells, in the CCs endothelial angiogenesis was ruled out by labeling each cell lineage separately, corroborating VM formation by TNBC cells. We believe that angiogenesis was not developed in the CCs due to the balance between specific factors induced by the physical interaction between ECs and cancer cells. In particular, the high abundance of anti-angiogenic TIMPs, together with the high plasticity of TNBC cells, may have overridden endothelial angiogenesis. Supporting this, in ECs, TIMP-2 has been shown to abolish proangiogenic factor-induced proliferation [43] and to disrupt FGF-2-stimulated mitogenesis [44]. Interestingly, in our in vitro model, VM was formed using two different TNBC cell lines, further corroborating that this malignant cell phenotype overrides EC-dependent angiogenesis when closely interacting. Thus, the cancer component more likely engages ECs into providing pro-VM factors. Among the tumor microenvironment cellular components affecting tumorigenesis, both SCs and ECs are relevant. However, only ECs were able to induce VM in the CCs, even though mesenchymal SCs are known to promote tumor angiogenesis and metastasis [45]. Therefore, we concentrated on studying EC–TNBC cell interaction for VM formation.

Regarding the plasticity of cancer cells, this feature is characteristic of stem cells that can go through VM, allowing for the transition into an endothelial-like phenotype, poor differentiation and metabolic reprogramming [5,6,8,35,46]. We were able to characterize several markers of these processes in our CCs. In particular, VE-cadherin, a critical adhesion molecule expressed in ECs and cancer cells able to perform VM [4,47], was found in the TNBC-VM structures. This finding is an important fact since VE-cadherin is known to promote VM [47]. For example, HER2 overexpression enabled MCF-7 breast cancer cells to form VM only if VE-cadherin protein was also overexpressed [48]. This evidence, together with our findings, suggest that VE-cadherin is a central link to VM formation regardless of the cell phenotype. Similarly, the status of HK2, an enzyme highly associated with metabolic reprogramming, was increased in the TNBC-VM segments. However, they were completely vimentin-negative, suggesting that the two TNBC cell lines tested herein were not going through epithelial-to-endothelial transition nor epithelial-to-mesenchymal transition when forming VM. Instead, they gained stemness markers, as observed via RT-qPCR increased gene expression of *MYC*, *OCT4*, *SOX2* and *KLF4*, otherwise known as the Yamanaka reprogramming transcription factors, and whose co-expression is sufficient to induce pluripotent stem cells [49]. Of note, several authors have pointed out that stem-like cancer cells are the ones responsible for VM formation and increased malignant behavior in distinct types of tumors [6,37,50]. Similar to our findings, the increase in stem cell markers’ gene expression in MCF-7 cells has been reported in a standardized protocol following indirect co-culture with ECs, although VM was not reported [42]. Altogether, this evidence further supports that the induction of the stem phenotype enables breast cancer cells to conform VM structures.

It is known that the activation of the angiogenic switch induces cellular metabolic and phenotypic changes [51,52]. So, we explored the secretome composition from EA.hy926 and TNBC cell monocultures and CCs. We found that angiogenesis-associated modulators were upregulated in the CCs, while some anti-angiogenic ones were downregulated. For example, I-TAC, also known as CXCL11, has been shown to exert anti-angiogenic/angiostatic properties through its binding to the CXCR3 receptor [53,54]. In our CCs, I-TAC abundance was reduced as compared to monocultures in the angiogenesis array. Interestingly, a study using the rabbit cornea micropocket model found that CXCR3 agonists were able to inhibit the angiogenic activity of IL-8 [55]. So, it is reasonable to believe that I-TAC downregulation favored IL-8 pro-vasculogenic effects in our CCs. Several molecules involved in angiogenesis are also related to VM induction [8,21]. One of them is in fact IL-8, which was among the most abundant factors in the CC’s secretome, as compared to monoculture’s CM. Notably, IL-8 inhibition has been reported to restrain VM in TNBC MDA-MB-231 cells [56]. Other upregulated factors in the CM of CCs included various cytokines, MMP1, MCP-1, ANG and PLGF, which have also been associated with VM and angiogenic processes [1,8,57].

Supporting the protein array, genes in ECs related to the angiogenic phenotype underwent transcriptional reprogramming when exposed to both the CM from CCs and from TNBC monocultures; for example, *FGFR1*, *VEGFR2* and *VEGFA* were upregulated, while *THBS1* was downregulated. Other authors have also shown transcriptional reprogramming of ECs when exposed to the secretome of tumor cells, for instance, the co-culture of glioma cells with primary human umbilical vein cells (HUVECs), resulting in their activation and the formation of net-like structures, secondary to the release of self-activating factors, such as FGF [39]. In addition, we observed via immunocytochemistry that FGFR1 and VEGFR2 were expressed in cancer cells engaged in VM, and intriguingly, they were also present in vesicles inside MMECs. This finding is consistent with other reports proposing extracellular vesicles and/or exosomes as microenvironment elements supporting tumor progression through favoring angiogenesis and cancer cell reprogramming [58,59,60]. Indeed, extracellular vesicles can contain as cargo a variety of angiogenic factors, such as ANG, IL-6, IL-8 and VEGF, or even mRNA, which can be incorporated by cells of the tumor microenvironment, changing their behavior [61]. For instance, VEGF-enriched exosomes released from ECs after an anti-angiogenic treatment significantly promoted VM in hepatocellular carcinoma [62]. Altogether, these pieces of evidence support the cross-talk between tumor cells and ECs to promote tumor vasculogenesis, warranting further research.

Interestingly, several studies have associated the formation of giant endothelial cells with pathological vascular conditions. These cells can arise following exposure to different agents, physical damage or metabolic stress [63,64,65,66]. Some of these cells have been named multinucleated variant endothelial cells, which possess phagocytic activity and have been associated with atherosclerosis severity [64]. In the oncological context, tumor-associated endothelial cells can acquire cytogenetic abnormalities induced by the tumor microenvironment, with intrinsic differences depending on the type of tumor they relate to [67,68]. Like MMECs in our study, giant endothelial cells have been shown to undergo rapid cell death, creating spaces containing subendothelial material within [63]. In the study by Tullossand and Booyse, the microfilamentous material remaining after EC death was thought to provide sites for platelet–vessel wall interactions and subsequent thromboembolic complications [63]. Although speculative, MMECs found in our CCs seemed to support vasculogenic mimicry formation, through providing both pro-VM factors as well as physical support, which deserves to be further investigated.

As it is known, and as shown herein, one of the mechanisms involved in the VM capacity of TNBC cells is the gaining of stemness features. Therefore, considering that FGF–FGFR signaling is highly involved in the maintenance of stemness and metabolic reprogramming in cancer cells [69,70], we hypothesized that FGFR could be a main driver of VM in our in vitro model. Moreover, in malignant glioma cells, the pharmacological inhibition of FGFR has proved to be sufficient for VM impairment, while the suppression of FGF signaling impaired the metabolism of ECs [15,71,72]. So, we explored the possibility of blocking VM in TNBC cells using an FGFR inhibitor. First, we were able to show that FGFR1 was expressed not only in the TNBC cells engaged in VM but also in the ECs and MMECs neighboring the branches of the cancer networks, strongly suggesting the involvement of this receptor in intercellular communication during VM. Remarkably, the blockade of FGFR signaling in the CCs using AZD4547 completely prevented TNBC tube-like network formation. This result is in line with very recent reports showing that Receptor Interacting Protein Kinase 1 (RIPK1)-dependent necroptosis promoted VM formation in TNBC cells [73], while AZD4547 potently inhibited necroptosis through selectively targeting RIPK1 [74]. Similarly, AZD4547 has been shown to inhibit stemness features in the BT-474 breast cancer cell line, including *MYC* gene expression [70], providing additional rationale for using AZD4547 to avoid VM formation. In addition, in our study, AZD4547 was able to downregulate several pro-VM/angiogenic factors, as shown in the protein array, while it upregulated I-TAC accumulation. Notably, as reported previously [53,54] and as discussed earlier in this paper, I-TAC has antiangiogenic/angiostatic effects, while high levels of this chemokine have been shown to exert antitumor immunity in breast cancer [75]. Nevertheless, its impact on VM formation has not yet been studied and deserves further research.

Finally, knowing that PI3K–Akt is involved in VM activation [10], we pharmacologically inhibited this signaling pathway with LY294002 and found that VM was suppressed, further suggesting that the FGFR/PI3K–Akt axis was a main regulator of VM in our CCs. Other works have previously shown that FGFR or PI3K/Akt inhibition can impair both angiogenesis and VM in different types of cancer, and that the pan-FGFR inhibitor PD173074 impaired VM in cultured TNBC cells [38,71,76,77,78]; however, to our knowledge, this is the first report of VM inhibition in breast cancer by AZD4547 and LY294002, warranting future clinical studies. Of note, phase 1 and phase 2 studies have shown that AZD4547 is active and relatively well tolerated in patients with solid tumors, including breast cancer [79,80].

Some limitations of our study include the lack of tridimensional confocal microscopy studies, which would have strengthened the data on protein expression and tube formation, and the lack of viability assays, since AZD4547 has previously shown to affect breast cancer cells proliferation and stemness features [70,81]. Another limitation is that, despite being one of the most widely used and well-characterized human vascular endothelial cells [82], EA.hy926 cells have been artificially immortalized, and as so, they can differ from unmodified ECs in some features. However, normal ECs and tumor-associated ECs are also structurally and functionally different. Regarding this, tumor ECs are known to acquire microenvironment-induced cytogenetic abnormalities, and to possess large, heterogeneous nuclei [67], a feature that we observed in the ECs from our study, thus probably reflecting more an advantage than a limitation. Moreover, EA.hy926 cells were created by fusing primary HUVECs with A549 cancer cells. Since breast cancer cells have been shown to form hybrids occasionally through fusing with tumor ECs [83], EA.hy926 cells may more accurately represent tumor-associated EC behavior. Notably, as judged via the cell tracker analyses, ECs and TNBC cells in our co-culture model did not fuse, allowing us to study their paracrine interaction in the context of VM.

In summary, herein we show that the bidirectional cross-talk between ECs and TNBC resulted in the formation of a proangiogenic microenvironment involving a mixture of cytokines and growth factors promoting VM via the PI3K–Akt signaling pathway, that can be readily inactivated by AZD4547 and LY294002. Thus, our results demonstrate the therapeutic potential of AZD4547 and LY294002 for VM prevention in breast cancer. The co-culture system used herein may serve as a valuable tool to further study endothelial-dependent VM formation as well as potential therapeutic strategies to abrogate it.

## 4. Materials and Methods

### 4.1. Reagents

Trizol was acquired from Life Technologies, Carlsbad, CA, USA. LightCycler TaqMan Master was from Roche (Roche Applied Science, Indianapolis, IN, USA). The reverse transcription (RT) system (Maxima™ Reverse Transcriptase) was from Thermo (Thermo-Scientific, St. Louis, MO, USA). The compound LY294002 in solution was from Calbiochem, La Joya, CA, USA, while AZD4547 (AstraZeneca, Cambridge, UK) was acquired from Santa Cruz (Santa Cruz Biotechnology Inc., Dallas, TX, USA).

### 4.2. Cell Cultures

We used two different TNBC cell lines: MBCDF-T, which was derived from an invasive ductal breast carcinoma primary cell culture [84,85], and the other one acquired from the American Type Culture Collection (HCC1806-CRL-2335, repository of cell lines from “Programa de Investigación en Cáncer de Mama” Universidad Nacional Autónoma de México). For the co-culture models, we used the human SC endometrial cell line N30, donated by Dr. Robert Taylor (Department of Obstetrics and Gynecology, Wake Forest School of Medicine, Winston Salem, NC, USA), as well as the human endothelium cell line EA.hy926 (American Type Culture Collection CRL-292). All cell lines were maintained in supplemented medium [DMEM-F12 medium, 100 units/mL penicillin, 100 μg/mL streptomycin, 5% fetal bovine serum (FBS)].

#### 4.2.1. Mixed CCs

We used mixed CCs to assess the role of the stroma and endothelium in the VM capacity of TNBC cells. Two cell lineages were simultaneously seeded: ECs + TNBC or SCs + TNBC cells (1:1 ratio, 250,000 each cell line) on glass coverslips placed into 6-well plates and incubated with 2 mL of supplemented medium under standard culture conditions. Control cultures with single cell lines were also undertaken. Bright-field images were captured in each case. After 48 h of culture, cells were fixed and processed to further study tube formation and specific VM markers expression via fluorescent immunocytochemistry as described below. The cell culture media obtained after these ECs/TNBC incubations were collected and stored at −70 °C for further use in the angiogenesis array. To know which cell line was conforming the VM structures in the EC/TNBC cells CCs, each cell lineage was independently labeled with a fluorescent green cell tracker following the manufacturer’s instructions (CellTracker™ Green CMFDA, Invitrogen, Thermo Fisher Scientific, Waltham, MA, USA) and co-seeded with the other unlabeled cell lineage. After 2 days, cells were washed and fixed in 80% ethanol for 10 min. After air drying, a drop of UltraCruz™ mounting medium containing 4′,6-diamidino-2-phenylindole (DAPI, Santa Cruz Biotechnology, Santa Cruz, CA, USA) was added to coverslips, which were placed onto glass slides and photographed with a conventional fluorescence microscope (DP72 camera, Olympus Optical Co., Ltd., Tokyo, Japan).

#### 4.2.2. Incubation of ECs with CM from CCs and Gene Expression Studies

To obtain insight into the gene expression changes induced by the secretome of binomial CCs on regulators of angiogenesis, we exposed confluent EA.hy926 cells to the CM obtained from mixed CCs (EC + TNBC cells) for 24 h. Total RNA was extracted from ECs using Trizol reagent. Then, two micrograms of RNA were reverse transcribed using the Maxima Reverse Transcriptase system, following the manufacturer’s instructions. The resultant cDNAs were used in qPCR analysis. Amplifications were carried out in the LightCycler^®^ 480 from Roche (Roche Diagnostics, Mannheim, Germany), according to the following protocol: activation of TaqDNA polymerase and DNA denaturation at 95 °C for 10 min, followed by 45 amplification cycles comprising 10 s at 95 °C, 30 s at 60 °C and 1 s at 72 °C. The primer sequences (upper/lower) and corresponding universal probe numbers (Roche, Germany) in parentheses are as follows: for *THBS1,* caatgccacagttcctgatg/tggagaccagccatcgtc (56); *FGFR1,* agactccggcctctatgctt/aggaggggagagcatctga (66); *HK2,* tcccctgccaccagacta/tggacttgaatcccttggtc (54); *VEGFR2,* gctcaagacaggaagaccaa/ggtgccacacgctctag (27)*; VEGFA,* ctacctccaccatgccaagt/ccacttcgtgatgattctgc (29). As an internal control, we used the gene expression of the housekeeping gene glyceraldehyde-3-phosphate dehydrogenase *GAPDH*, agccacatcgctgagacac/gcccaatacgaccaaatcc (60).

### 4.3. Immunocytochemistry

Mixed CCs of ECs with TNBC cells were allowed to interact for 48 h. Then, the cells were fixed in ice-cold 80% ethanol for 10 min and permeabilized with perm/wash buffer [1X Phosphate-buffered saline (PBS) pH 7.4, 3% FBS, 0.05% Tween 20) for 15 min. Perm/wash buffer was also used for antibodies dilutions. Washing steps were performed with wash buffer (1X PBS pH: 7.4, 1% FBS, 0.05% Tween 20). Coverslips were incubated overnight at 4 °C, pairing one anti-rabbit and one anti-mouse primary antibody as indicated in corresponding figure legends. The following antibodies were used: rabbit anti-HIF1A (1:300, Abcam, Cambridge, MA, USA), mouse anti-Vimentin (1 μg/mL, Abcam), rabbit anti-VEGFR-2 (1:200, Cell Signaling Technology, Danvers, MA, USA), mouse anti-FGFR1 (1:60, Santa Cruz Biotechnology) or rabbit anti-FGFR1 (1:200, Cell Signaling), mouse anti-HK2 (1:100, Santa Cruz Biotechnology) and mouse anti-VE-Cadherin (1 μg/mL, BioLegend, San Diego, CA, USA). Slides were washed and further incubated with goat anti-mouse-Cyanine3 (Cy3) antibody (1:1000, Life Technologies Inc., Carlsbad, CA, USA) and goat anti-rabbit-Fluorescein isothiocyanate (FITC, 1:500, Jackson ImmunoResearch Laboratories, West Grove, PA, USA) for 2 h at room temperature. After washing, one drop of mounting medium for nucleus visualization was applied to the coverslips, which were then placed onto slides. Cells were photographed with a conventional fluorescence microscope.

### 4.4. Angiogenesis Antibody Array

In order to identify the presence of activators and inhibitors of the vasculogenic process in the CM, we used a human angiogenesis antibody array in membranes format (ab193655, Abcam). This assay displays antibodies spotted on the membranes for 43 different targets. All steps were carried out as recommended by the manufacturer. Briefly: after blocking, the membranes were incubated overnight at 4 °C in the presence of 1 mL undiluted CM from either monocultured ECs, HCC1806, MBCDF-T or co-cultured ECs + HCC1806, ECs + MBCDF-T, ECs + HCC1806 + AZD4547 5 μM, or ECs + MBCDF-T 5 μM. After washing, incubations were performed with a biotinylated-secondary antibody cocktail followed by the HRP-conjugated streptavidin mixture (2 h each at room temperature). Chemiluminescence signals were analyzed in a ChemiDoc XRS+ System (BioRad, Hercules, USA). Semi-quantitative comparisons between samples were performed with the image acquisition and analysis software Image Lab, version 4.0.1 (BioRad) using the positive controls included in each membrane. Antigen spots without signal were disregarded. A semi-supervised hierarchical analysis of the targets was carried out in GraphPad Prisma v7. software.

### 4.5. Evaluation of AZD4547 and LY294002 Effects on VM Morphometric Parameters

To quantitatively evaluate the effect of AZD4547 and LY294002 on the VM capacity of TNBC cells, cells were co-cultured with EA.hy926 in the presence or absence of 5 μM AZD4547 or 6 μM LY294002 for 48 h. Bright-field images were acquired in VM hot spots via conventional microscopy. Two different observers counted the segments and meshes per visual field in hot spots of at least three 4× images. As segments, we considered cords or branches formed by TNBC cells, delimited by two junctions, while meshes were spotted as closed areas surrounded by segments.

### 4.6. Statistical Analysis

In RNA expression studies, the Mann–Whitney rank sum test was used when the normality test failed; otherwise, Student’s *t*-test was used for each gene studied. Data were expressed as the mean ± standard error of the mean (SEM). Statistical significance was considered at *p* < 0.05.

## 5. Conclusions

Co-culturing EA.hy926 ECs with TNBC cells induced in vitro VM formation through molecular reprogramming and phenotypic cellular transformation via activating the FGFR/PI3K/Akt axis. VM formation was triggered via the proangiogenic signature produced by EA.hy926 ECs when they interacted physically with TNBC cells in co-culture, a process efficiently inhibited by AZD4547 or LY294002. These findings contribute to our understanding of the vasculogenic processes associated with tumorigenesis and hold the potential for enhancing cancer treatment strategies through targeting endothelial-dependent VM induction.

## Figures and Tables

**Table 1:** Relative gene expression of OSKM genes.

Gene	Control	+CM
*OCT4*	3.24 × 10^−8^ ± 3.2 × 10^−8^	8.28 × 10^−5^ ± 3.8 × 10^−5^ *
*SOX2*	0.00	6.89 × 10^−4^ ± 6.7 × 10^−4^ *
*KLF4*	0.00	2.48 × 10^−6^ ± 3.9 × 10^−6^ *
*MYC*	1.14 × 10^−3^ ± 5.4 × 10^−4^	5.27 × 10^−3^ ± 1.7 × 10^−3^

The gene expression is depicted as relative to that of GAPDH. CM = conditioned media from CCs. N = three different experiments with triplicate replicates each. * denotes *p* < 0.05 vs. control.

## Data Availability

The authors confirm that the data supporting the findings of this study are available within the article [and/or] its Supplementary Materials.

## Supplementary Materials

The following supporting information can be downloaded at: https://www.mdpi.com/article/10.3390/ijms241813770/s1.
