# Supplementary material for: The Inhibition of the FGFR/PI3K/Akt Axis by AZD4547 Disrupts the Proangiogenic Microenvironment and Vasculogenic Mimicry Arising from the Interplay between Endothelial and Triple-Negative Breast Cancer Cells
Source: Int J Mol Sci. 2023 Sep 6;24(18):13770. doi: 10.3390/ijms241813770 (PMC10531243; doi:10.3390/ijms241813770)
Supplement: Supplementary file 1 [file ijms-24-13770-s001.zip › Figure S1.pdf]

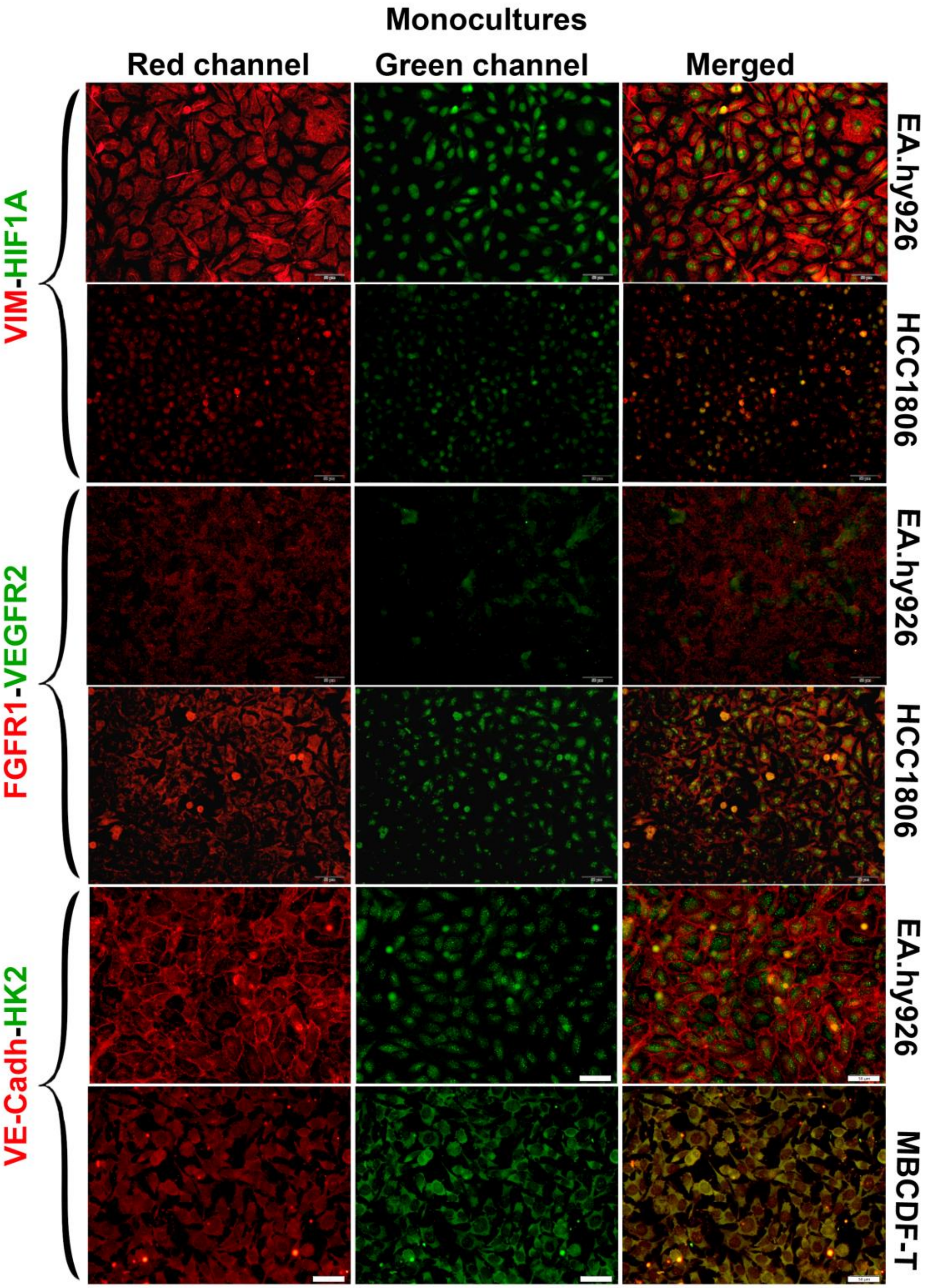

Figure S1. Monocultured TNBC cells and endothelial cells differentially express markers of VM and endothelium. HCC1806, MBCDF-T or EA.hy926 cells were cultured alone for 48 h. Then, cells were fixed and processed for immunocytochemistry. Two primary antibodies were used for each slide, one made in mice and one in rabbits. Incubations were undertaken overnight at 4°C. All mice-made primary antibodies were identified using a secondary goat anti-mouse-Cyanine3 (Cy3) antibody (red channel, left panels), while all rabbit-made antibodies were tagged using a goat anti-rabbit-Fluorescein isothiocyanate (FITC, green channel, middle panels). The right panels show merged pictures. Cells were photographed with a conventional fluorescence microscope. Representative 20x images are shown. Vimentin (VIM); hypoxia-inducible factor-1α (HIF1); VE-Cadherin (VE-Cadh); Hexokinase 2 (HK2), Vascular endothelial growth factor receptor 2 (VEGFR2); Fibroblast growth factor receptor 1 (FGFR1).
