# Supplementary material for: The Inhibition of the FGFR/PI3K/Akt Axis by AZD4547 Disrupts the Proangiogenic Microenvironment and Vasculogenic Mimicry Arising from the Interplay between Endothelial and Triple-Negative Breast Cancer Cells
Source: Int J Mol Sci. 2023 Sep 6;24(18):13770. doi: 10.3390/ijms241813770 (PMC10531243; doi:10.3390/ijms241813770)
Supplement: Supplementary file 1 [file ijms-24-13770-s001.zip › Supplementary Table S1.pdf]

	Monocultures			Cocultures		+ AZD4547	
Factor	HCC1806	MBCDF-T	EC	EC+HCC	EC+MB	EC+HCC	EC+MB
IL-6	0.00	0.00	1.41E-01	5.45E-02	2.02E-01	3.63E-02	1.13E-01
IL-2	8.52E-03	0.00	0.00	1.49E-02	3.42E-02	0.00	0.00
IL-1a	2.59E-02	2.86E-02	0.00	4.18E-02	4.50E-02	2.72E-02	1.99E-02
IL-1b	1.74E-02	0.00	0.00	2.64E-02	6.23E-02	9.37E-03	1.15E-02
IL-8	1.85E-03	0.00	2.40	3.56	2.57	1.93	2.92
IL-4	1.43E-02	7.14E-03	2.27E-02	0.00	3.51E-02	0.00	1.76E-03
MCP-1	0.00	0.00	1.67	3.03	1.96	1.34	1.91
MCP-3	1.52E-02	0.00	0.00	0.00	1.21E-01	2.71E-02	4.60E-02
GRO	1.50E-05	1.19E-03	0.00	7.91E-03	0.00	1.90E-02	3.36E-02
IL-10	0.00	0.00	0.00	2.15E-02	3.03E-02	4.45E-02	1.84E-03
I-TAC	1.46E-02	0.00	1.12E-02	0.00	3.63E-04	4.68E-02	6.76E-03
EGF	1.64E-03	0.00	3.57E-03	0.00	1.94E-02	4.37E-03	0.00
VEGF	0.00	0.00	0.00	5.55E-02	4.98E-02	2.47E-03	1.03E-03
bFGF	7.59E-03	0.00	1.06E-02	6.09E-02	0.00	0.00	0.00
PLGF	0.00	0.00	0.02	0.12	0.28	0.21	0.28
TIMP-1	0.00	0.00	0.24	0.43	0.30	0.16	0.29
TIMP-2	4.96E-03	0.00	2.04	3.23	2.11	1.31	2.39
MMP1	1.40E-02	0.00	0.00	3.74	6.10	0.74	7.13
ANG	3.38E-03	0.00	1.53E-01	4.98E-02	1.79E-01	1.22E-01	1.65E-01
ANGPT1	1.83E-02	3.86E-03	0.00	5.68E-02	7.54E-02	2.38E-02	7.14E-02
ANGPT2	8.61E-02	5.53E-02	4.32E-02	8.93E-02	1.09E-01	6.06E-02	2.98E-01
PECAM	1.42E-02	0.00	0.00	1.51E-02	2.81E-02	0.00	5.87E-02
TIE-2	1.22E-02	0.00	3.16E-03	5.14E-03	5.13E-02	0.00	2.04E-02
ENA78	4.05E-03	0.00	1.20E-02	1.08E-02	3.30E-02	2.09E-02	0.00

Supplementary Table S1. The interaction between endothelial and TNBC cells induces a pro-angiogenic secretome, which is suppressed by AZD4547. Results from the angiogenesis antibody array analysis showing the factors detected in the secretome (conditioned media, CM) from monocultured endothelial cells EA.hy926 (EC), HCC1806 (HCC) or MBCDF-T (MB) cells, as well as from the CM of co-cultures of ECs with each TNBC cell line (EC+HCC or EC+MB), either alone or in the presence of AZD4547 (5 μM) incubated for 48 h. The antibody-containing membranes were incubated overnight at 4°C in the presence of 1 mL undiluted CM. Semi-quantitative comparisons between samples were performed using the positive controls included in each membrane. Antigen spots without signals were disregarded. The threshold of 0.01 was used to consider the most abundant factors in the samples.
